# Production of Bioactive Peptides from Baltic Herring (*Clupea harengus membras*): Dipeptidyl Peptidase-4 Inhibitory, Antioxidant and Antiproliferative Properties

**DOI:** 10.3390/molecules27185816

**Published:** 2022-09-08

**Authors:** Sari Mäkinen, Jaakko Hiidenhovi, Xin Huang, Amanda dos Santos Lima, Luciana Azevedo, Jari Setälä, Anna-Liisa Välimaa, Pirjo Mattila, Daniel Granato

**Affiliations:** 1 Production Systems Unit—Natural Resources Institute Finland (Luke), FI-31600 Jokioinen, Finland; 2 Faculty of Nutrition, Federal University of Alfenas, Rua Gabriel Monteiro da Silva, 714, Alfenas 37130-000, Brazil; 3 Production Systems Unit—Natural Resources Institute Finland (Luke), FI-20520 Turku, Finland; 4 Production Systems Unit—Natural Resources Institute Finland (Luke), University of Oulu, FI-90014 Oulu, Finland; 5 Bioactivity and Applications Lab, Department of Biological Sciences, Faculty of Science and Engineering, University of Limerick, V94 T9PX Limerick, Ireland

**Keywords:** bioactive peptides, antioxidants, antidiabetic effects, cell-based experiments, reactive oxygen species

## Abstract

This study aimed to produce bioactive protein hydrolysates from undervalued fish, namely Baltic herring, and its filleting by-products. Protein hydrolysates were produced with Alcalase and Flavourzyme to achieve effective hydrolysis. The hydrolysates were evaluated for chemical composition, molecular weight distribution, antioxidant capacity, dipeptidyl-peptidase 4 (DPP4) inhibitory activity, effects on cell proliferation and surface hydrophobicity. The protein content of the hydrolysates was high, from 86% to 91% (dm), while the fat content was low, from 0.3% to 0.4% (dm). The hydrolysates showed high DPP4 inhibition activities with IC_50_ values from 5.38 mg/mL to 7.92 mg/mL. The scavenging activity of the hydrolysates towards DPPH was low, but an intermediate Folin–Ciocalteu reducing capacity and Cu^2+^ chelating ability was observed. The solid phase extraction with Sep-Pak C18 cartridges increased the DPP4 inhibition activity and antioxidant capacity, indicating peptides’ crucial role in the bioactivities. The cytotoxicity of the hydrolysates was evaluated on the HCT8, IMR90, and A549 cell lines. The hydrolysates inhibited cell growth in the cancer and normal cells, although they did not reduce cell viability and were not lethal. Overall, our results indicate that protein hydrolysates from Baltic herring have potential as health-promoting foods and nutraceuticals, especially for enhancing healthy blood glucose regulation.

## 1. Introduction

Baltic herring (*Clupea harengus membras*) is a highly underutilized fish resource in the Baltic Sea area. It comprises the main catch of commercial fishing, with an annual catch slightly below 100 million kg, but only 15% of the catch is used for human consumption [1]. The Finnish Baltic herring fishery was awarded an Marine Stewardship Council (MSC) certificate in 2018, recognizing its sustainable and environmentally friendly fishing practices. Baltic herring fishing also removes a significant amount of eutrophic nutrients from the Baltic Sea every year [2,3]. However, the value of this fish reserve remains low, as most of the catch is used for fur animal feed or fishmeal factories. In the filleting industry, by-products such as head, fins, skin, frame and bones comprise approximately 60% of the total weight of the fish [4]. In the case of Baltic herring, filleting produces about 2 million kilos of by-products annually. The filleting of Baltic herring is concentrated in a few places, so the by-products of the filleting industry are logistically easy to utilize.

The value of the commercial Baltic herring catch could be multiplied if the small fishes and filleting by-products could be used as high-value products. Recent scientific research has revealed the potential of fish by-products as a source of protein hydrolysates and bioactive peptides [5,6]. For example, peptides with dipeptidyl peptidase IV (DPP4) inhibitory activity have been produced from rainbow trout (*Oncorhynchus mykiss*) by-products [7], Atlantic salmon (*Salmo salar)* skin [5] and Sardine (*Sardina pilchardus*) [8], proposing their application as functional ingredients for preventing and controlling type-2 diabetes. There is a high commercial potential and industrial interest in producing protein hydrolysates and bioactive peptides from fish by-products. However, challenges remain, especially in verifying health claims and ensuring the sensory quality of the products.

Regarding the industrial applications of peptide-rich fractions, the antioxidant capacity in biological media plays a significant role: it is known that antioxidants (e.g., phenolics, carotenoids, peptides, and others) should be bioavailable and should not be cytotoxic to normal human cells (IMR90) [9]. Considering these factors, the evaluation of the antioxidant capacity using different chemical-based methods, such as the use of 1,1-diphenyl-2-pycril-hydrazil (DPPH) radical, ferric reducing antioxidant power (FRAP), Folin–Ciocalteu reducing capacity, and the reducing power constitute a suitable screening assessment of the bioactivity potential of peptide-rich fractions [10]. More importantly, the antioxidant capacity of peptide-rich fractions and their effects on the human cell growth and proliferation should also be assessed to safeguard their toxicologically safe utilization in food applications. In practice, it is important to cover a range of cell morphology, such as epithelial (HCT8 and A549) and fibroblast (IMR90), and to assay the compounds under cancer and normal cells, which possess specific growth, metabolic and genomic patterns. Additionally, when dealing with food materials, the gut cells should be observed once they are at the first site of contact with any compound in the body during the digestion process [11].

The present study aimed to apply proteolysis by food-grade commercial enzymes to produce bioactive peptides from whole Baltic herrings and Baltic herring filleting by-products. The recovered protein hydrolysates were evaluated for cell-based antioxidant capacity and DPP4 inhibitory activities. Peptide fractions were also characterized for their molecular weight profile, proximate composition and effects on cell proliferation.

## 2. Results and Discussion

### 2.1. Chemical Characterization of the Hydrolysates

The present study used a combination of Alcalase and Flavourzyme to achieve the extensive hydrolysis of Baltic herring protein. Alcalase is a high-spectrum, non-specific endo-peptidase that mainly binds to hydrophobic amino acids. It is widely used to produce bioactive peptides as it results in the hydrophobic characteristics of the residues and increases the availability of N-terminal sites [12]. Flavourzyme is a complex blend of endo and exo-peptidases providing unique flavor generation and debittering benefits. Its effectiveness, particularly in animal protein applications, bases its use after an endo-protease treatment to generate small peptides [13].

All samples of the present study showed high protein content varying from 60% (Baltic herring side stream, BHSS) to 91% (Baltic herring side stream hydrolysate, BHSSH) (Table 1). The hydrolysis treatment resulted in a remarkable increase in the protein content; the protein hydrolysate of whole Baltic herring (BHH) protein was 21% points higher in comparison to the raw materials (BH). The difference in the Baltic herring side stream was even higher; the hydrolysate (BHSSH) protein content was 31% points higher than in the side stream without hydrolysis treatment (BHSS). The increase in the protein content was mainly due to the efficient separation of fat in the hydrolysis process; whilst the fat content in the hydrolysates (BHSSH and BHH) was close to zero (Table 1).

**Figure 1:**
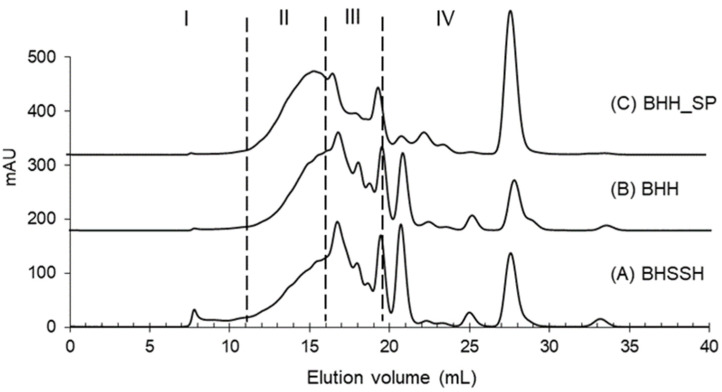
Size exclusion chromatograms of Baltic herring hydrolysates. A = Baltic herring side stream hydrolysate (BHSSH); B = Baltic herring hydrolysate (BHH); and C = Sep-Pak purified Baltic herring hydrolysate (BHH_SP). The molecular weight distribution was divided into four ranges I: >10,000 Da; II: 10,000–1000 Da; III: 1000–200 Da; and IV: <200 Da.

Molecular weight distribution, as deduced from SEC chromatograms (Figure 1), showed that small compounds with a molecular weight below 1 kDa comprised the significant fraction of hydrolysates, namely BHH and BHSSH (Table 2). The most prevalent fraction in both hydrolysates was 0.2–1 kDa, while the share of compounds larger than 10 kDa was merely insignificant (Table 2). The Sep-Pak purified Baltic herring hydrolysate showed a partly different molecular weight distribution, with the most prevalent fraction being 1–10 kDa. Solid-phase extraction with the Sep-Pak cartridges diminishes non-protein compounds, and hence, the difference is presumably due to the small molecular weight of non-protein compounds such as minerals and possible sugar moieties present in the crude hydrolysates. However, all hydrolysates contained a large portion, 20–30%, of small compounds with an MW less than 0.2 kDa. This fraction corresponds to small peptides (di- and tripeptides) and free amino acids. Generally, bioactive peptides suitable for the formulation of therapeutic foods are primarily below 1 kDa in molecular weight [14]. The molecular weight distribution results show that the Alcalase and Flavourzyme treatment extensively hydrolyzed the Baltic herring proteins into small, <1 kDa, molecular weight peptides.

### 2.2. Antioxidant and DPP4 Inhibitory Capacity In Vitro

The antioxidant activity of the hydrolysates and their capacity to inhibit the DPP4 activity are shown in Table 3. The bioactivities were measured from crude hydrolysates and after solid-phase extraction with Sep-Pak cartridges (BHH_SP) in the case of the hydrolysate prepared from the whole Baltic herrings. In the antioxidant activity analysis, the Sep-Pak purified hydrolysate (BHH_SP) presented the highest (*p* < 0.05) mean values for the Folin–Ciocalteu reducing capacity, free-radical scavenging activity towards DPPH, and chelating ability towards Cu^2+^. The crude hydrolysates without Sep-Pak purification (BHH and BHSSH) showed intermediate Folin–Ciocalteu reducing capacity and Cu^2+^ chelating ability, while the scavenging activity towards DPPH was very low. BHSSH presented the lowest (*p* < 0.05) reducing power, but with the other antioxidant assays, there were no statistical differences between the crude BHH and BHSSH samples (*p* > 0.05).

Similarly, Durand et al. [15] used herring milt to obtain hydrolysates (MW < 800 Da) processed with a mix of enzymes, with the subsequent ultrafiltration and found a 2.7-fold increase in the oxygen radical absorption capacity (ORAC) compared to the unhydrolyzed fraction. Pacific herring (*Clupea pallasii*) protein was hydrolyzed by Wang et al. [16], and three fractions were obtained: protein hydrolysate PHPH-I (MW > 10,000 Da), PHPH-II (MW = 3500–10,000 Da) and PHPH-III (MW < 3500 Da) and the antioxidant activity of these fractions were assessed using the DPPH and the hydroxyl radical scavenging assays. Fraction PHPH-I presented the lowest (*p* < 0.05) antioxidant activity in both antioxidant activity assays, while sample PHPH-III presented the highest DPPH value. Samples PHPH-II and PHPH-III showed similar (*p* > 0.05) scavenging activity towards the hydroxyl radical, but PHPH-III had a higher cellular antioxidant effect in HepG-2 cells. The fresh farmed rainbow trout (*Oncorhynchus mykiss*) by-product (36% head, ~27% viscera, ~7% fins and ~30% backbone) was hydrolyzed using Alcalase and fractionated using different chemical processes [17]. They found that the different hydrolysate fractions presented antioxidant activity not only using the FRAP (ferric reducing antioxidant power; 100–200 µmol Trolox/g) and ABTS (2,2′-azino-bis(3-ethylbenzothiazoline-6-sulfonic acid; 300–1300 µmol Trolox/g) assays, but also Fe^2+^ chelating ability (20–70 µmol EDTA/g). According to Nikoo et al. [17], peptides with a lower MW have a higher negative charge (carboxyl groups)-to-mass ratios than peptides with a higher MW, enabling them to form complexes with metal ions more efficiently.

Regarding DPP4 inhibition, the highest efficacy was shown by the BHH_SP (Table 3). This Sep-Pak-purified hydrolysate presented the lowest (*p* < 0.05) IC_50_ value, indicating that 5.38 mg/mL of the sample is sufficient to inhibit 50% of the DPP4 activity. Crude hydrolysates BHH and BHSSH showed similar DPP4 inhibitory activities with no statistical difference (*p* > 0.05) (Table 3). Both hydrolysates inhibited the activity of the DPP4 enzyme effectively; the obtained IC_50_ values were 7.50 mg/mL and 7.92 mg/mL for BHSSH and BHH, respectively. The surface hydrophobicity of the hydrolysates was in the same trend as the DPP4 inhibitory activity (Table 3). After Sep-Pak purification, the surface hydrophobicity significantly increased because the Sep-Pak column was packed with C_18_ material, which binds the peptides and proteins by hydrophobic interactions. The DPP4 inhibitory peptides are generally rich in hydrophobic amino acids, and the presence of hydrophobic amino acids enhances the interaction with the hydrophobic pocket in the DPP4 [18]. Protein raw materials with a high content of hydrophobic amino acids are thus a suitable substrate for producing DPP4 inhibitory peptides. For example, proline-rich proteins, wheat gluten, gelatin, and collagens (skins from pigs and fish) have been studied for producing DPP4 inhibitory peptides [18]. The DPP4 inhibitory activities of the Baltic herring hydrolysates are comparable to those of salmon skin gelatin hydrolysates [19].

### 2.3. In Vitro Assays of Cytotoxicity and Proliferation

**Figure 2:**
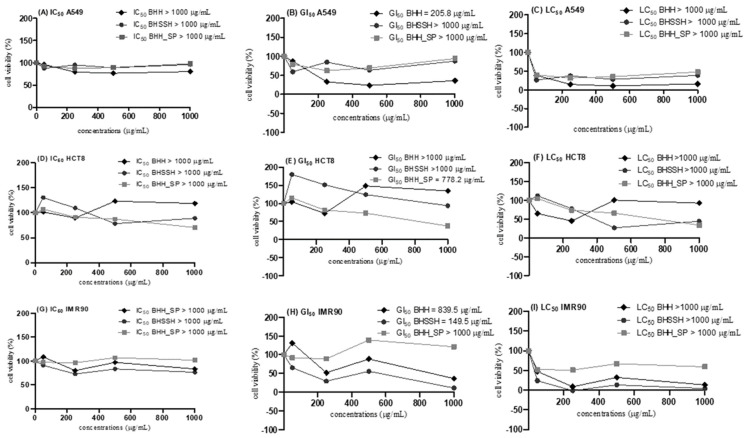
Cell viability and proliferation evaluation of the concentration-dependent effect after 48 h exposure to BHH, BHSSH and BHH_SP extracts in IMR90, A549 and HCT8 cell lines. (**A**,**D**,**G**) IC_50_ (concentration of the extracts that inhibits cell viability by 50%); (**B**,**E**,**H**) GI_50_ (concentration of the extracts inhibits cell growth by 50%); and (**C**,**F**,**I**) LC_50_ (concentrations of the extracts that result in the loss of 50% cells).

The cytotoxicity of the hydrolysates from the whole Baltic herring and side streams was evaluated in different concentrations on HCT8, IMR90 and A549 cell lines. Figure 2 shows the antiproliferative and cytotoxicity effects of the samples BHSSH (Baltic herring side stream hydrolysate), BHH (Baltic herring hydrolysate) and BHH_SP (Sep-Pak purified Baltic herring hydrolysate).

The results show that the hydrolysates from whole Baltic herring and side streams inhibit cell growth (IG_50_) in both cancer and normal cells, although they do not reduce cell viability (IC_50_) and are not lethal (LC_50_). BHSSH, BHH and BHH_SP promote an antiproliferative effect in IG_50_ concentrations between 149.5 and 839.5 μg/mL after 48 h of treatment. Similarly, short peptides from the heated products of half-fin anchovy have shown antiproliferative activity against human prostate cancer PC-3 cells, although in higher concentrations (5–40 mg/mL) [20]. Additionally, red tilapia-derived hydrolysates have been reported to promote cell growth at higher concentrations (2 mg/mL) after 24 h of treatment [21].

Among the samples, the BHH and BHH_SP demonstrated a more significant antiproliferative effect on lung (A549 IG_50_ = 205.8 μg/mL) and intestinal cells (HCT8 IG_50_ = 778.2 μg/mL), respectively, and were more effective on the cancer cell compared to the normal cell. On the other hand, the BHSSH showed a more antiproliferative effect in normal lung fibroblast (IG_50_ = 149.5 μg/mL) and did not have the same effect on cancer cells (A549 and HCT8). Contrary to these results, Himaya et al. [22] found that a purified peptide from Pacific cod hydrolysate did not show any significant toxicity toward normal lung fibroblast (MRC-5) at the tested concentration (10, 25 and 50 µM). According to Halim et al. [23], the antiproliferative capacity of the peptides may be related to their hydrophobicity, molecular weight and antioxidant capacity. Our hydrolysates contain various mixtures of small molecular weight peptides. Thus, a comparison with the results of a single purified peptide sequence is not straightforward.

The presence of specific shorter peptides can increase molecular mobility, and diffusivity and exert direct cytotoxicity on cancer cells through membrane rupture and penetration. Additionally, they can enhance interaction with the cell components [11,24]. The molecular weight and amino acid composition of the peptides (e.g., Lys and Arg) influence the antiproliferative capacity [18]. The low molecular weight and high content of hydrophobic peptides at the N-terminus may increase peptide antiproliferative activity. However, it is challenging to identify amino acids that give optimum antiproliferative activity due to limited studies [11,25,26].

Overall, our cytotoxicity and proliferation results highlight an antiproliferative behavior of Baltic herring hydrolysates against lung cells. The results are consistent with the chemical analysis, where BHH and BHSSH presented a higher protein content after hydrolysis and increased the share of low molecular weight peptides. Therefore, these samples exerted more antiproliferative activity than the other samples. This effect is hypothesized by Picot et al. [27], who approaches a binding competition between fish peptides and FCS growth factors on cell membrane receptors. Fish peptides could act as antagonists of FCS growth factor receptors, but no clear correlation was determined between the degree of hydrolysis and antiproliferative activity. According to Heffernan et al. [26], further work on the isolation, identification and elucidation of the mechanism of action of fish-derived anti-cancer peptides is suggested.

## 3. Materials and Methods

### 3.1. Chemical Reagents and Cell Lines

Gallic acid, ascorbic acid, Folin–Ciocalteu phenol reagent, pyrocatechol violet (3,3′,4-trihydroxyfuchsone-2″-sulfonic acid), 1-anilino-8-napthalenesulfonat (ANS) were purchased from Sigma-Aldrich (Helsinki, Finland). 2,2-Diphenyl-1-picrylhydrazyl radical (DPPH) was purchased from Alfa Aesar (Ward Hill, MA, USA). Potassium hexacyanoferrate (III) was obtained from Merck (Darmstadt, Germany). Gly-Pro-p-nitroanilide was purchased from VWR (Helsinki, Finland). Dipeptidyl peptidase IV enzyme, Sitagliptin, Alcalase^®^, Flavourzyme^®^, ovalbumin, α-lactalbumin, aprotinin, vitamin B12 and Na-hippuric acid were purchased from Merck (Espoo, Finland). 3-4,5 dimethylthiazol-2, 5 diphenyl tetrazolium bromide (MTT), Dulbecco’s Modified Eagle’s Medium/Nutrient Mixture F-12 Ham (DMEM) were purchased from Sigma-Aldrich (São Paulo, Brazil). The other reagents were of analytical grade, and the solutions were prepared with ultrapure water. Normal human lung fibroblast (IMR90), lung adenocarcinoma epithelial cells (A549) and human ileocecal adenocarcinoma cells (HCT8) were obtained from the Rio de Janeiro Cell Bank (Rio de Janeiro, Brazil).

### 3.2. Fish Samples

Baltic herring filleting by-products were obtained, and whole Baltic herrings were purchased from Martin Kala (Turku, Finland). In this study, both fish samples (whole Baltic herrings (BH) and Baltic herring filleting by-products (BHSS)) were hydrolyzed using Alcalase and Flavourzyme in combination. First, both fish raw materials were ground using a household meat grinder (Bosch, Germany). Subsequently, the fish mince was mixed with water (1:1, *w*/*w*), stirred with an overhead stirrer and heated to 55 °C, after which the hydrolysis was initiated by adding Alcalase (Enzyme/Substrate (E/S) = 2% *v*/*v*). Hydrolysis was performed for 3 h at 55 °C, and consequently, Flavourzyme (E/S = 1%) was added, and the hydrolysis was continued for 1 h. The hydrolysis reaction was terminated by heating (85 °C for 15 min), after which the hydrolysate was centrifuged (10,000× *g*, 20 min) to separate soluble and insoluble fractions. Supernatants were further filtrated using the Buchner funnel equipped with two Whatman 40 filter papers with a layer of Celite between them. The obtained liquid fraction was freeze-dried and stored at −20 °C for further analysis.

Before the in vitro analysis, the supernatants of the hydrolysates prepared from the whole Baltic herrings were pre-treated with solid phase extraction using Sep-Pak^TM^ cartridges to diminish the interference of non-protein compounds. Furthermore, in the text, BHH refers to hydrolysates prepared from the whole Baltic herring and BHSSH to the hydrolysates prepared from the Baltic herring side streams.

### 3.3. Proximate Composition

The moisture and ash contents in samples were determined using TGA701 Thermo-gravimetric Analyser (Leco Corporation, St. Joseph, MI, USA). TGA measured the weight loss as a function of temperature (105 °C and 650 °C for moisture and ash, respectively) under controlled conditions and automatically determined the moisture and ash contents in samples with the selected program. The nitrogen content was determined with an in-house Kjeldahl method based on the International Organization for Standardization methods [28,29] using a Kjeltec TM8400 analyzer (Foss Analytical Ab, Höganäs, Sweden). A conversion factor of 6.25 was used to calculate the total protein content. The total fat content was determined using the SoxCap TM 2047 in combination with the Soxtec TM 2050 extraction system (Foss A/B, Hillerød, Denmark) with a preparatory acid hydrolysis step and diethyl ether extraction according to the International Organization for Standardization [30].

### 3.4. Antioxidant Activity In Vitro

The scavenging effect of the DPPH radical (5–45 mg/L, R^2^ = 0.997) was evaluated using the methodology described by Brand-Williams et al. [31] and the results were expressed as mg AAE/g. Folin–Ciocalteu reducing capacity (11–150 mg/L, R^2^ = 0.999) was assessed using the methodology described by Margraf et al. [32] and the results were expressed as mg GAE/g. The Cu^2+^ chelating ability (7.5–100 mg/L, R^2^ = 0.996) was evaluated using pyrocatechol violet as the chromogen agent and the results were expressed as mg of disodium ethylenediaminetetraacetic acid (EDTA) per gram of material, (mg EDTAE/g) [33]. The reducing power (5–40 mg/L, R^2^ = 0.999) was assessed using the Prussian Blue assay according to the methodology outlined by Margraf et al. [32] and the results were expressed as mg GAE/g. All analyses were conducted in quadruplicate.

### 3.5. DPP4 Inhibition In Vitro

DPP4 inhibitory activity was measured in triplicate according to the method described by Lacroix and Li-Chan [34] with some modifications. Briefly, hydrolyzed Baltic herring samples were diluted to the target concentrations in 100 mM Tris-HCl buffer pH 8.0. In a 96-well microplate, 25 µL of diluted sample was pre-incubated with 25 µL of substrate Gly-Pro-p-nitroanilide (1.52 mM) at 37 C for 30 min, after which 50 µL of DPP4 enzyme (0.01 Units/mL) was added, and the mixture was incubated at 37 °C for 30 min. The reaction was stopped by adding 100 µL of 1 M Na-acetate buffer, pH 4.0, and the absorbance of the samples was measured at 405 nm using Perkin Elmer Wallac 1420 Victor2 Microplate Reader (PerkinElmer, Turku, Finland). The DPP4 inhibition was defined as the percentage of DPP4 activity inhibited by a given hydrolysate concentration (protein basis).

The IC_50_ values (concentrations of hydrolysate required to cause a 50% inhibition of the DPP4 enzyme activity) were determined from the cubic regression equations generated by fitting the data from the plot of % of DPP4 inhibition against hydrolysate concentrations ranging from 2.5 to 10 mg/mL (final assay concentration, protein basis). The experiments were conducted in triplicate, and sitagliptin was used as a reference inhibitor.

### 3.6. Surface Hydrophobicity of the Fish Hydrolysates

The surface hydrophobicity of the hydrolysates was determined by the 1-anilino-8-napthalenesulfonat (ANS) binding assay [35]. The hydrolysates were diluted by 50 mM potassium phosphate buffer (pH 7.4) to protein concentrations of 0.015–0.0015% (*w*/*v*). The protein concentration was according to Table 1. The diluted hydrolysate solution (1 mL) was mixed with 5 µL of 8 mM ANS in the same buffer in a 96-well microplate (black-walled, clear-bottom) and the fluorescent intensity of the ANS-hydrolysates mixture using excitation wavelength at 390 nm (bandwidth 9 nm) and emission wavelength at 470 nm (bandwidth 20 nm) (Tecan Infinite M200, Männedorf, Switzerland). The fluorescent intensity was plotted against the protein concentration by linear regression analysis, and the initial slope S0 was calculated and represented as surface hydrophobicity. Three replicates were performed for each fish hydrolysate.

### 3.7. In Vitro Assays of Cytotoxicity and Proliferation

The cytotoxicity and proliferation assays of peptides from Baltic herring fish and by-products were determined by the MTT (3-(4,5-dimethylthiazol-2-yl)-2,5-diphenyl tetrazolium bromide) assay. The IMR90 (human lung fibroblast), A549 (lung adenocarcinoma epithelial cells) and HCT8 (human colon carcinoma) were obtained from the Rio de Janeiro cell bank (Rio de Janeiro, Brazil) and seeded in 96-well plates at a density of 8 × 10³ (IMR90) and 1 × 104 cells/well (A549 and HCT8), 100 μL/well, in Dulbecco’s Modified Eagles’ Medium/Nutrient Mixture F-12 Ham (DMEM) supplemented with a heat-inactivated fetal bovine serum to finals concentration of 20% (IMR90) and 10% (A549, HCT8). Cells were allowed to adhere for 24 h at 37 °C in a humidified atmosphere with 5% CO2. After 24 h of adherence, the medium was removed. The peptides from Baltic herring hydrolysates (BHH and BHSSH) at different concentrations ranging from 50 to 1000 (μg/mL) were added to the cells in a 100 μL culture medium. After 48 h exposure, the medium was removed, and the MTT was added to each well and incubated for 4 h. After 4 h, MTT was removed, 100 μL of DMSO was added to each well, the plate was shaken for 10 min to solubilize formazan crystals, and absorbance was read at 570 nm. The absorbance was measured at 570 nm using a microplate reader (SynergyTM H1, Biotek) and Gen5TM data analysis software. The experiments were conducted in quadruplicate, and the dose-response analysis was determined by nonlinear regression (curve fit) using GraphPad Prism^®^ (GraphPad Software, Inc., San Diego, CA, USA) software. According to the method described by Do Carmo et al. [36], the IC50, GI50 and LC50 parameters were calculated.

### 3.8. Evaluation of Molecular Weight Distribution by Size Exclusion Chromatography (SEC)

Size exclusion chromatograms of hydrolysates were determined with Äkta Basically Bettered liquid chromatographic system (GE Healthcare Life Sciences, Uppsala, Sweden) equipped with Superdex Peptide HR 10/30 column (GE Healthcare Life Sciences, Uppsala, Sweden). Peptides were dissolved in 100 mM sodium phosphate buffer (pH 7) and eluted with the same buffer at a 0.5 mL/min flow rate. The eluate was monitored by a UV detector at 214 nm.

A molecular weight calibration curve was prepared from the average elution volume of the following standards: ovalbumin (43,000 Da), α-lactalbumin (14,178 Da), aprotinin (6511 Da), vitamin B12 (1355 Da) and Na-hippuric acid (201 Da). The total surface area of the chromatograms was integrated and separated into four ranges (>10,000, 1000–10,000, 200–1000, <200 Da), and the results are expressed as a percentage of the total area.

### 3.9. Statistical Analyses

Experiments were conducted at least three times, and the results were expressed as a means followed by the standard deviation. One-way analysis of variances (ANOVA) was used to detect any significant (*p* < 0.05) differences between the samples, and Tukey’s test was conducted to differentiate the means. TIBCO Statistica v. 13.3 (TIBCO Software Ltd., Palo Alto, CA, USA) was used in the analyses.

## 4. Conclusions

In the present study, we used undervalued fish, namely Baltic herring, and its filleting by-products, for producing bioactive protein hydrolysates with DPP4 inhibitory and antioxidant activities and antiproliferative effects. Hydrolysates were produced with food-grade proteases Alcalase and Flavourzyme. The recovered hydrolysates were high in protein, low in fat and contained small MW peptides, <1 kDa, over 50% of the total protein. The chemical antioxidant capacity of the hydrolysates varied from intermediate to low, but the inhibition activity against DPP4 was highly effective. The removal of non-protein compounds by Sep-Pak C18 extraction increased the bioactivities of the hydrolysate, indicating the crucial role of peptides in DPP4 inhibition and antioxidant activity. The Baltic herring hydrolysates inhibited cell growth in lung cells, although they did not reduce cell viability and were not lethal. Our results indicate that Baltic herring and its by-products can be processed into protein hydrolysates with the potential for promoting health, especially healthy blood glucose regulation. Studies to assess the effects of the hydrolysates in vivo, unveiling the mechanisms of action and linking the bioactivity with the MS data, are needed in the future to proceed towards product applications.

## Figures and Tables

**Table 1:** Proximate composition of Baltic herring raw materials and hydrolysates (*n* = 3).

Composition (g/100 g DM)	BHSS	BHSSH	BH	BHH
Protein	60.2 ± 0.4 ^c^	91.2 ± 1.2 ^a^	67.1 ± 2.5 ^b^	86.1 ± 1.0 ^a^
Fat	23.3 ± 0.5 ^a^	0.40 ± 0.05 ^b^	24.0 ± 0.8 ^a^	0.30 ± 0.04 ^b^
Ash	16.6 ± 0.9 ^a^	8.1 ± 0.1 ^b^	8.9 ± 3.2 ^b^	4.2 ± 0.8 ^c^

Note: BHSS = Baltic herring side stream; BHSSH = Baltic herring side stream hydrolysate; BH = Whole Baltic herring; BHH = Whole Baltic herring hydrolysate; Different superscript letters represent statistically different (*p* < 0.05) results.

**Table 2:** Proportion (%) of molecular weight distributions of Baltic herring hydrolysates.

	I (%) >10,000 Da	II (%) 10,000–1000 Da	III (%) 1000–200 Da	IV (%) <200 Da
BHSSH	4.0 ± 0.2 ^a^	29.5 ± 0.8 ^c^	40.1 ± 0.8 ^b^	26.4 ± 0.3 ^b^
BHH	0.9 ± 0.2 ^b^	32.0 ± 0.7 ^b^	43.3 ± 1.0 ^a^	23.6 ± 0.5 ^c^
BHH_SP	1.1 ± 0.3 ^b^	40.8 ± 0.9 ^a^	27.2 ± 1.1 ^c^	30.8 ± 0.4 ^a^

BHSSH = Baltic herring side stream hydrolysate; BHH = Baltic herring hydrolysate; and BHH_SP = Sep-Pak purified Baltic herring hydrolysate. Different superscript letters represent statistically different (*p* < 0.05) results.

**Table 3:** Antioxidant, DPP4 inhibition, and surface hydrophobicity properties of Baltic herring hydrolysates.

Hydrolysates	Reducing Power (mg GAE/g)	Folin–Ciocalteu Reducing Power (mg GAE/g)	DPPH (mg AAE/g)	Cu^2+^ Chelating Ability (mg EDTAE/g)	DPP4 Inhibition Activity (IC_50_: mg/mL)	Surface Hydrophobicity (S0)
BHSSH	0.07 ± 0.01 ^c^	28.51 ± 0.57 ^b^	0.29 ± 0.02 ^b^	21.01 ± 0.08 ^b^	7.50 ± 0.03 ^a^	4430 ± 250 ^a^
BHH	0.18 ± 0.00 ^a^	29.11 ± 0.32 ^b^	0.08 ± 0.03 ^c^	21.32 ± 0.09 ^b^	7.92 ± 0.16 ^a^	4530 ± 480 ^a^
BHH_SP	0.16 ± 0.01 ^b^	36.43 ± 0.68 ^a^	8.84 ± 0.53 ^a^	27.08 ± 0.03 ^a^	5.38 ± 0.27 ^b^	18260 ± 2130 ^b^

Note: BHSSH = Baltic herring side stream hydrolysate; BHH_SP = Sep-Pak purified Baltic herring hydrolysate; BHH = Baltic herring hydrolysate; GAE = gallic acid equivalents; EDTAE = EDTA equivalents; AAE = ascorbic acid equivalents. Different superscript letters represent statistically different (*p* < 0.05) results.

## Data Availability

Not applicable.

## Sample Availability

Samples of the Baltic herring hydrolysates are available from the authors.
